# Clinical pattern of liver injury in drug reaction with eosinophilia and systemic symptoms (DRESS): a retrospective study in Taiwan

**DOI:** 10.1186/2045-7022-4-S3-P18

**Published:** 2014-07-18

**Authors:** Kai-Lung Chen, I-Chun Lin, Hong-Chih Yang, Chia-Yu Chu

**Affiliations:** 1 National Taiwan University Hospital, Department of Dermatology, Taiwan; 2 National Taiwan University Hospital, Department of Internal Medicine, Taiwan

## Background

Drug reaction with eosinophilia and systemic symptoms (DRESS) is a syndrome with multi-systemic involvements. Liver injury is the most common visceral manifestation (80%).

## Method

To investigate the types of liver injury and factors involved, including the chronology, recovery time, relationship with culprit drugs and the pathology correlations, a retrospective study was conducted by reviewing all medical records of DRESS patients diagnosed between Dec. 2000 and Jan. 2013 in the National Taiwan University Hospital Database. DRESS was defined according to the International Registry of Severe Cutaneous Adverse Reactions (RegiSCAR). The pattern of liver damage is classified according to the International Consensus Meeting criteria.

## Results

72 cases were retrieved. Among them, 62 (86.1%) cases had liver injury, with 6 (9.7%) patients having liver injury before skin presentation. We found 23 patients were cholestatic type (35.5%), following by 17 patients with mixed type (29.0%) and 12 patients of hepatocelluar type (19.4%). Compared with those without liver injury, peripheral atypical lymphocytes were more commonly seen in patients with liver injury (74.2% and 30.0%, p=0.01), but the amount of eosinophils did not show significant difference. Patients with hepatocellular type were relatively young, with the mean age of 36.6 (p=0.03). More patients in hepatocellular type (83.3%) had extreme value of liver injury, comparing with cholestatic and mixed types (39.1% and 58.8%, p=0.042). Patients of the extreme group were younger, with the mean age of 40.6 (p=0.006). When comparing with non-extreme group, they tended to have fever (90.9% and 65.5%, p=0.026), took more time to recover (percentage of recovery less than 30 days, 24.2% and 48.3%, p=0.065) and had less eosinophils on skin biopsy (30.8% and 88.9%, p=0.001).

## Conclusions

Liver injury may be a pro-drome of DRESS. Peripheral atypical lymphocytosis can be a predictor of liver injury. Younger patients are more in hepatocellular type, with higher liver function tests, relatively slow recovery, and less eosinophils on skin biopsy.

